# Integrin signalling regulates YAP and TAZ to control skin homeostasis

**DOI:** 10.1242/dev.133728

**Published:** 2016-05-15

**Authors:** Ahmed Elbediwy, Zoé I. Vincent-Mistiaen, Bradley Spencer-Dene, Richard K. Stone, Stefan Boeing, Stefanie K. Wculek, Julia Cordero, Ee H. Tan, Rachel Ridgway, Val G. Brunton, Erik Sahai, Holger Gerhardt, Axel Behrens, Ilaria Malanchi, Owen J. Sansom, Barry J. Thompson

**Affiliations:** 1 The Francis Crick Institute, 44 Lincoln's Inn Fields, London WC2A 3LY, UK; 2 The Beatson Institute, Switchback Rd, Bearsden, Glasgow G61 1BD, UK; 3 Edinburgh Cancer Research Centre, University of Edinburgh, Western General Hospital, Crewe Road South, Edinburgh EH4 2XR, UK

**Keywords:** Hippo pathway, Integrin, Yes-associated protein, TAZ, Stratified squamous epithelium

## Abstract

The skin is a squamous epithelium that is continuously renewed by a population of basal layer stem/progenitor cells and can heal wounds. Here, we show that the transcription regulators YAP and TAZ localise to the nucleus in the basal layer of skin and are elevated upon wound healing. Skin-specific deletion of both YAP and TAZ in adult mice slows proliferation of basal layer cells, leads to hair loss and impairs regeneration after wounding. Contact with the basal extracellular matrix and consequent integrin-Src signalling is a key determinant of the nuclear localisation of YAP/TAZ in basal layer cells and in skin tumours. Contact with the basement membrane is lost in differentiating daughter cells, where YAP and TAZ become mostly cytoplasmic. In other types of squamous epithelia and squamous cell carcinomas, a similar control mechanism is present. By contrast, columnar epithelia differentiate an apical domain that recruits CRB3, Merlin (also known as NF2), KIBRA (also known as WWC1) and SAV1 to induce Hippo signalling and retain YAP/TAZ in the cytoplasm despite contact with the basal layer extracellular matrix. When columnar epithelial tumours lose their apical domain and become invasive, YAP/TAZ becomes nuclear and tumour growth becomes sensitive to the Src inhibitor Dasatinib.

## Abstract (precis)

**Summary:** In the squamous epithelium of the mouse skin, the transcription factors YAP and TAZ are regulated by integrin-Src signalling, and are required for proliferation of skin stem/progenitor cells.

## INTRODUCTION

The Yes-associated protein (YAP) family of transcriptional co-activators are emerging as potent oncoproteins that strongly drive cell proliferation in many types of stem/progenitor cells and cancers (Harvey et al., 2013; Irvine and Harvey, 2015; Pan, 2010, 2015; Piccolo et al., 2013). The function of YAP family co-activators was first discovered by *Drosophila* genetics, where the sole YAP homologue Yorkie (Yki) was found to be necessary and sufficient to promote cell proliferation and tissue overgrowth in epithelia (Huang et al., 2005). Subsequent genetic experiments in mice showed that ectopic expression of YAP (also known as YAP1) was sufficient to drive cell proliferation in liver, intestine, bronchus and skin (Cai et al., 2010; Camargo et al., 2007; Dong et al., 2007; Schlegelmilch et al., 2011; Zhang et al., 2011a; Zhao et al., 2014). Surprisingly, YAP knockout mice have mild phenotypes, although they are deficient in proliferative repair of the intestine and resistant to intestinal tumour formation (Azzolin et al., 2014; Cai et al., 2010), as well as showing reduced bronchial stem cells (Zhao et al., 2014) and kidney defects (Reginensi et al., 2015). An important and widespread physiological role for YAP in mice might be obscured by the possibility of redundancy between YAP and TAZ (also known as WWTR1) a second mammalian family member that is highly similar in both sequence and function.

At the molecular level, Yki and YAP were shown to function by associating with the DNA-binding transcription factor Scalloped (Sd; or TEAD in humans) to drive transcription of anti-apoptotic and pro-proliferative target genes (Koontz et al., 2013; Liu-Chittenden et al., 2012; Vassilev et al., 2001; Wu et al., 2008). Other co-factors of Yki/YAP that promote transcription include WBP2 (Zhang et al., 2011b), MASK1/2 (Sansores-Garcia et al., 2013; Sidor et al., 2013) and the SWI/SNF complex (Jin et al., 2013; Oh et al., 2013). The activity of Yki was found to be regulated by the *Drosophila* Hippo-Warts (Hpo-Wts) kinase signalling pathway, in which Wts directly phosphorylates Yki to promote its relocalisation from the nucleus to the cytoplasm (Dong et al., 2007; Huang et al., 2005; Oh and Irvine, 2008). In human cells in culture, YAP nuclear localisation is similarly inhibited upon LATS1/2 kinase phosphorylation, because phosphorylated YAP is retained the cytoplasm by binding to 14-3-3 family proteins (Dong et al., 2007; Zhao et al., 2007). This entire molecular system is now referred to as the Hippo signalling pathway.

Much recent work has aimed to identify upstream regulators of Hippo signalling. A group of apically localised proteins including Crumbs (Crb, CRB1/2/3 in humans), Merlin (Mer, NF2 in humans), Expanded (Ex, similar to Willin and AMOT in humans) and Kibra (Kib, KIBRA or WWC1 in humans) were found to activate Hippo signalling (repressing Yki activity) in *Drosophila* epithelia (Baumgartner et al., 2010; Chen et al., 2010; Genevet et al., 2010; Hamaratoglu et al., 2006; Ling et al., 2010; Varelas et al., 2010; Yu et al., 2010) and in mice (Szymaniak et al., 2015). In addition, a group of adherens junction-localised proteins including Ajuba (Jub), Zyxin (Zyx), Dachs, Mib and Riquiqui (Riq), were shown to inhibit Hippo signalling (activating Yki) in *Drosophila* epithelia (Cho et al., 2006; Das Thakur et al., 2010; Degoutin et al., 2013; Gaspar et al., 2015; Mao et al., 2006; Rauskolb et al., 2011). Finally, manipulation of the level of F-actin in *Drosophila* can also affect Hippo signalling, possibly via signalling through the Src kinase, which can promote Yki activation (Enomoto and Igaki, 2013; Fernandez et al., 2011, 2014; Sansores-Garcia et al., 2011). Human YAP and TAZ were subsequently found to act as F-actin responsive mechanosensors in cell culture (Aragona et al., 2013; Benham-Pyle et al., 2015; Dupont et al., 2011; Zhao et al., 2007), but how their subcellular localisation is physiologically regulated in human epithelial tissues and cancers *in vivo* remains a fundamental unsolved problem.

Here, we examine the physiological function and regulation of YAP and TAZ in mammalian epithelial tissues. We focus on stratified squamous epithelia, particularly the skin, and compare our findings with columnar epithelia, such as the intestine and bronchus. We propose that YAP and TAZ act as sensors of both apical and basal signals *in vivo*, and that this regulatory logic explains why these proteins localise to the nucleus in basal stem/progenitor cells to promote cell proliferation and tissue renewal. Elevation of YAP and TAZ can then drive increased cell proliferation during wound healing or tumour formation.

## RESULTS

### YAP and TAZ are expressed in both mouse and human skin, and regulate gene expression in basal layer stem/progenitor cells

**Fig. 1.**
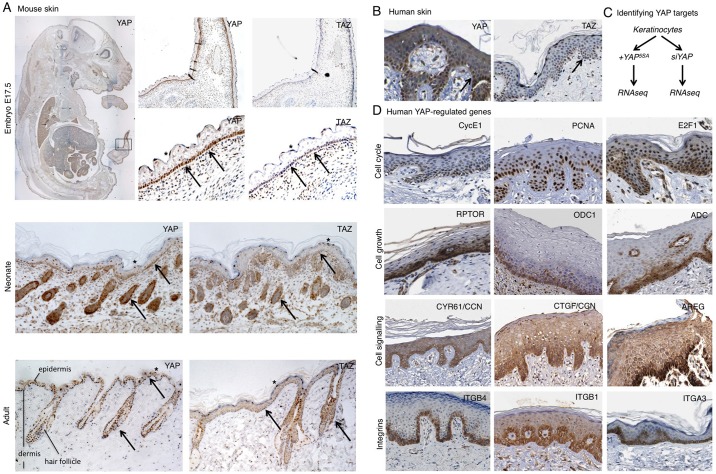
**YAP and TAZ are expressed in both mouse and human skin and regulate gene expression in basal layer stem cells.** (A) Mouse skin at three developmental stages, including embryonic (E17.5), neonate and adult. Tissue sections were stained for either YAP or TAZ to reveal their expression and subcellular localisation. (B) Human skin (adult) stained for either YAP or TAZ. Note the nuclear localisation in basal layer stem/progenitor cells as well as terminally differentiating flattened cells. Other differentiating cells have cytoplasmic YAP and TAZ localisation. Arrows indicate nuclear YAP/TAZ; asterisks indicate flattened suprabasal cells with nuclear YAP/TAZ. (C) Analysis of YAP-dependent gene expression by RNA-seq was performed by comparison of YAP gain and loss of function in keratinocytes (see Fig. S1). (D) YAP-regulated genes identified by RNA-seq analysed for their expression patterns in skin tissue by mining the Human Protein Atlas dataset (see Materials and Methods). Strong enrichment in basal layer stem/progenitor cells is evident for many target genes, indicating that YAP and TAZ are transcriptionally active in this population of cells.

We began by characterising the expression and subcellular localisation of YAP and TAZ in both mouse and human skin. Both proteins were found to be expressed and nuclear localised in a subset of cells in the skin of the mouse embryo, neonate and adult. Nuclear localisation of YAP and TAZ was particularly prominent in basal layer cells of both interfollicular epidermis and the hair follicle (Fig. 1A). Some nuclear localisation was also detected in the highly flattened squamous cells, consistent with results in cell culture where cell flattening induces nuclear accumulation of YAP and TAZ (Dupont et al., 2011) (Fig. 1A). Human YAP and TAZ show a similar pattern of subcellular localisation in sections of adult human skin (Fig. 1B). Basal layer cells feature nuclear YAP and TAZ, whereas differentiating daughters feature cytoplasmic YAP and TAZ (Fig. 1B). Again, some nuclear localisation is also detectable in highly flattened squamous cells that have terminally differentiated (Fig. 1B).

To confirm that YAP and TAZ are transcriptionally active in the skin, we sought to identify YAP-regulated genes by an RNA-sequencing (RNA-seq) approach in human keratinocytes. mRNA was isolated from cells expressing activated mutant *YAP^5SA^* or siRNAs against *YAP* and subjected to RNA-seq and gene-set enrichment analysis (Fig. 1C, Fig. S1). We found that the YAP-regulated gene sets included: the previously identified Hippo/YAP reactomes; cell cycle reactomes (such as E2F targets or cyclin E-associated genes); cell growth reactomes (such as Myc, global translation regulators or regulation of ornithine decarboxylase); cancer signalling reactomes (such as EGFR-Ras signalling targets); and cancer microenvironment/metastasis reactomes (including regulators of cellular interactions with the extracellular matrix) (Fig. S1). We therefore analysed the expression of the corresponding cell cycle [CycE1 (CCNE2), PCNA, E2F1], cell growth [RPTOR, ODC1, ADC (AZIN2)] and EGFR and integrin signalling (CYR61, CTGF, AREG, integrins α3, α6, β1, β2, β4) regulators (Fig. 1D). We found a striking restriction of these YAP targets to the basal layer of the skin, indicating that YAP/TAZ transcriptional regulation is active exclusively in the basal stem/progenitor cell population (Fig. 1D).

### YAP and TAZ are required for skin homeostasis

**Fig. 2.**
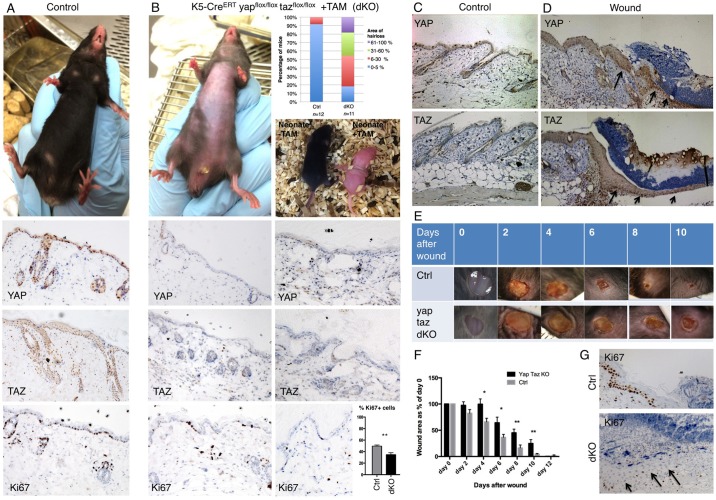
**Conditional inactivation of YAP and TAZ impairs skin homeostasis and wound repair in mice.** (A) Control mice have a thick layer of hair (fur) covering their skin, which sections reveal is positive for YAP, TAZ and Ki67 (a marker of cell proliferation). (B) Double conditional knockout mice for YAP and TAZ treated with tamoxifen as adults or neonates exhibit dramatic hair loss. Adult skin sections are negative for YAP and TAZ, and have reduced levels of Ki67^+^ positive cells (quantified as a percentage of total interfollicular basal cells in each randomly selected 40× field of view. *n*=757 control cells; *n*=896 dKO cells). (C) Control mouse skin stained for YAP and TAZ. (D) Punch biopsy wound edge stained for YAP and TAZ. (E) Imaging of wound healing in control (*n*=8) and YAP/TAZ double conditional knockout mice (dKO; *n*=8). Note delayed healing in dKO. (F) Quantification of wound healing rates in control versus dKO animals. ImageJ was used to measure the wound area at each stage. (G) Proliferation of cells as marked by Ki67 staining is reduced in dKO wounds versus control animals. Values are means±s.e.m. *P<0.05, **P<0.01.

To examine the physiological role of YAP and TAZ, we generated double conditional knockout (dKO) mice with the skin-specific Keratin5-CreERT recombinase. Compared with control animals, the YAP/TAZ dKO mice showed a dramatic loss of hair in patches beginning 2 weeks after tamoxifen injection in adult mice, or causing complete blockade of hair growth in neonates treated with tamoxifen (Fig. 2A,B). Histological sections of the skin revealed expression and nuclear localisation of YAP and TAZ in control skin, which was lost in the dKO tissue. Proliferation of basal layer cells, as marked by Ki67 staining, was clearly reduced in YAP/TAZ dKO skin (Fig. 2A,B), as was YAP target gene expression (Fig. S2). These phenotypes are reminiscent of skin-specific conditional knockouts of integrin β1 (ITGB1) (Brakebusch et al., 2000; Grose et al., 2002; Piwko-Czuchra et al., 2009; Raghavan et al., 2000; Singh et al., 2009).

We next tested whether YAP and TAZ contribute to skin repair after wounding. We found that levels of both YAP and TAZ were elevated after wounding, particularly in the basal cell layer of the epidermis where strong nuclear staining is visible (Fig. 2C,D). We next recorded the time taken to repair small (4 mm) wounds in the back skin of control versus YAP/TAZ dKO mice. We found that control wounds normally healed completely by 10 days, whereas dKO wounds failed to heal within 10 days and instead required an additional 2 days to heal (Fig. 2E,F). This delay in healing was not observed when YAP or TAZ were deleted individually. To investigate the cause of the delay in wound healing, we examined cell proliferation in wounds of control versus dKO mice. We found that the number of Ki67^+^ cells was reduced in dKO wounds versus controls (Fig. 2G). These findings demonstrate a crucial, physiological requirement for YAP and TAZ in basal layer stem/progenitor cells to promote cell proliferation.

In the skin of the ITGB1 conditional knockout mice, cells that escape Cre-mediated recombination are able to repopulate the mutant skin in a short time frame (Piwko-Czuchra et al., 2009). We found that the same phenomenon occurs in YAP/TAZ dKO skin, where after Cre activation and YAP/TAZ deletion, either YAP^+^ or TAZ^+^ residual cells expanded their territory, consistent with the notion that YAP and TAZ promote proliferation of basal layer cells (Fig. S3). This phenomenon was also evident during wound healing, where YAP or TAZ positive cells were able to populate the wound and allow proliferation and healing in dKO animals (Fig. S3). These findings underscore the importance of YAP and TAZ in epidermal progenitor cell proliferation and skin homeostasis and suggest a close relationship between integrin signalling and YAP/TAZ function.

### Mechanisms controlling nuclear localisation of YAP in basal layer cells

**Fig. 3.**
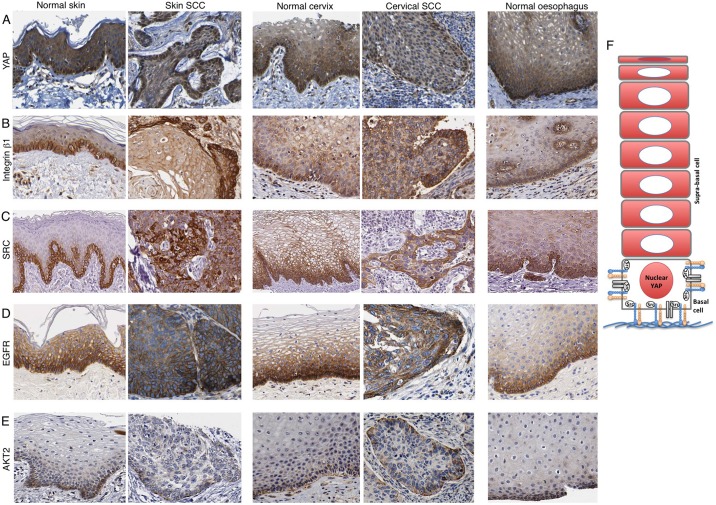
**Integrin-Src and EGFR-PI3K localisation in human stratified squamous epithelia and squamous cell carcinomas (SSCs).** The Human Protein Atlas dataset was mined to compare the expression and localisation of potential YAP regulators in human skin sections. YAP staining reveals basal layer nuclear localisation (A), ITGB1, SRC and EGFR staining reveals basal layer expression (B-D) and AKT2 staining reveals basal subcellular localisation (E) across squamous tissue types and cancers. (F) Model for YAP regulation in stratified squamous epithelia.

We next sought to understand how YAP and TAZ become nuclear localised in basal layer cells. Since YAP and TAZ are similar proteins that localise identically in skin, we henceforth focus on the regulation of YAP localisation. Recent work in cultured MCF10A breast cancer cells indicate a role for integrin-Src signalling and EGFR-PI3K signalling in promoting the nuclear localisation of YAP (Fan et al., 2013; Kim and Gumbiner, 2015). To test whether these pathways are active in skin, we examined their expression and subcellular localisation. By mining the Human Protein Atlas dataset, we found that ITGB1, SRC, EGFR and AKT2 (a marker of PI3K activation) are all expressed strongly in basal layer cells, with AKT2 recruited to the interface between basal layer epidermal cells and the underlying basement membrane extracellular matrix (Fig. 3A-E). This pattern is also evident in other squamous epithelia such as cervix or oesophagus and is retained in squamous cell carcinomas (Fig. 3A-E). These data suggest that nuclear YAP localisation might be stimulated by integrin-Src and/or PI3K signalling in basal layer skin keratinocytes (Fig. 3F).

**Fig. 4.**
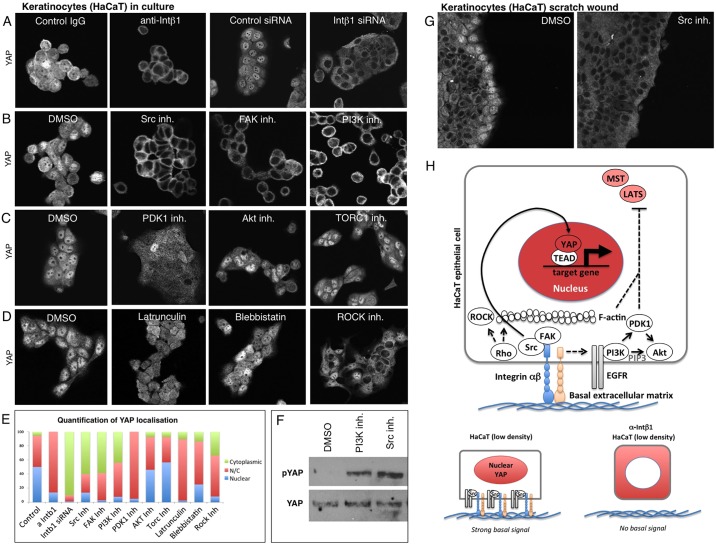
**Basal integrin-Src signalling promotes nuclear localisation of YAP in human HaCaT keratinocyte epithelial cells.** (A) YAP nuclear localisation is prevented by treatment of keratinocytes with anti-ITGB1 antibodies (PD52) or by *ITGB1* siRNA treatment. (B) YAP nuclear localisation is prevented by treatment of keratinocytes with the Src inhibitor Dasatinib, by the FAK inhibitor PF573228 or by the PI3K inhibitor GDC0941, but not by treatment with DMSO solvent. (C) YAP nuclear localisation is reduced by treatment of keratinocytes with the PDK1 inhibitor BX795, but not by the AKT inhibitor MK2206, TORC1 inhibitor Everolimus or DMSO solvent. (D) YAP nuclear localisation is reduced by treatment of keratinocytes with the F-actin destabilising drug Latrunculin, the myosin II inhibitor Blebbistatin, or the Rho-kinase inhibitor Y27632. (E) Quantification of A-D. (F) Western blotting analysis of p-YAP levels in keratinocytes treated with either DMSO control, PI3K inhibitor or Src inhibitor. Total YAP levels are shown as a control. (G) Nuclear YAP localisation at the leading edge of a scratch wound in keratinocyte culture is abolished by treatment with the Src inhibitor Dasatinib. (H) Schematic diagram of YAP regulation in keratinocytes.

To confirm that integrin-Src and PI3K signalling pathways are required for YAP nuclear localisation in keratinocytes, we systematically manipulated these pathways with siRNA knockdown or treatment with specific inhibitor compounds in human keratinocytes in culture. We found that inhibition of ITGB1 with blocking antibodies or siRNA, inhibition of the downstream effectors SRC or FAK, or inhibition of PI3K profoundly impairs YAP nuclear localisation (Fig. 4A,B). Interestingly, inhibition of the PI3K effectors AKT and TORC1 had no effect on YAP localisation, whereas inhibition of PDK1 did partially impair YAP nuclear localisation (Fig. 4C). Notably, drugs inhibiting F-actin, myosin II or Rho kinase had only a moderate effect on YAP localisation in keratinocytes (Fig. 4D). Quantification of these phenotypes highlights the strong effect of inhibition of integrin-Src and PI3K (Fig. 4E). Both Src inhibitors and PI3K inhibitors led to a clear increase in phosphorylated YAP (p-YAP), indicating that Hippo signalling (MST-LATS signalling) is elevated by these treatments (Fig. 4F). We confirmed these findings in a classic ‘scratch-wound’ assay, where Src inhibition completely reversed the nuclear localisation of YAP at the leading edge (Fig. 4G). These findings indicate that integrin-Src and EGFR-PI3K signalling are essential for YAP nuclear localisation in keratinocytes (Fig. 4H).

**Fig. 5.**
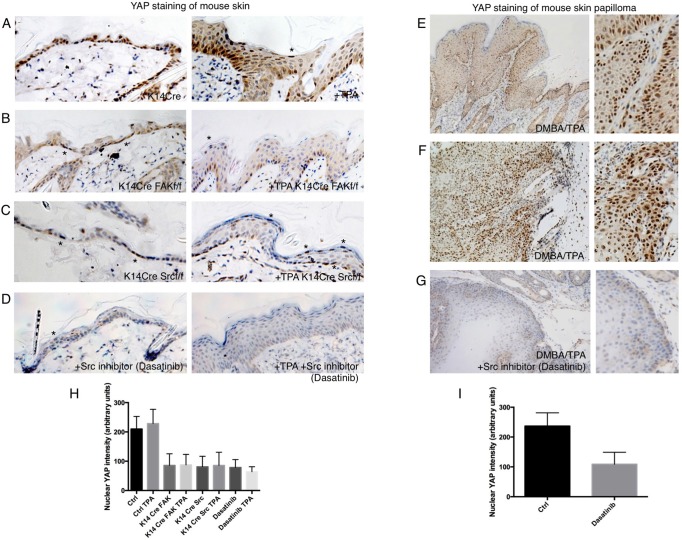
**Basal integrin-Src signalling promotes YAP stability and nuclear localisation in mouse skin.** (A) YAP staining in control and TPA-treated skin to induce hyperplasia. (B) YAP staining is reduced in FAK conditional KO skin before or after treatment with TPA. Note some residual nuclear YAP localisation in basal layer cells or highly flattened cells (asterisk). (C) YAP staining is reduced in Src conditional KO skin before or after treatment with TPA. Note that there is some residual nuclear YAP localisation in basal layer cells or highly flattened cells (asterisks). (D) YAP staining is reduced in Dasatinib-treated skin before or after treatment with TPA for 2 days. Note there is some residual nuclear YAP localisation in basal layer cells or highly flattened cells (asterisk). (E) YAP staining of mouse skin papilloma induced by DMBA-TPA treatment of mice expressing *v-Ha-Ras* (see Materials and Methods). Note stronger nuclear localisation in the basal layer. (F) YAP staining of mouse skin squamous cell carcinoma induced by DMBA-TPA treatment of *v-Ha-Ras*-expressing mice. (G) YAP staining is strongly reduced by treatment of DMBA-TPA induced papillomas with the Src inhibitor Dasatinib topically for 3 days. (H) Quantification of nuclear YAP intensity in A-D. (I) Quantification of nuclear YAP intensity in F,G. Values are means±s.e.m.

To extend these findings *in vivo*, we examined the role of integrin-Src signalling in mouse skin. We compared YAP localisation in untreated and TPA-treated (inflamed) skin samples from control animals and knockouts for FAK or Src (Fig. 5A-C). We found that loss of FAK or Src results in decreased YAP levels and nuclear localisation in both normal and inflamed skin (Fig. 5A-C). Some nuclear YAP remained in flattened cells (Fig. 5A-C, asterisks). Similar results were obtained by treatment of mice with the topical Src inhibitor (Dasatinib), which was able to drastically reduce YAP levels and nuclear localisation in untreated or TPA-treated skin, as well as in skin papillomas induced by a TPA+DMBA treatment regimen (Fig. 5D-I). These findings show that integrin-Src signalling is crucial to promote YAP stabilisation and nuclear localisation in basal layer stem/progenitor cells. Accordingly, recent work indicates that skin papillomas induced by DMBA+TPA in mice can be strongly reduced in size and frequency by homozygous deletion of YAP along with one copy of TAZ or by treatment with Dasatinib (Creedon and Brunton, 2012; Serrels et al., 2009; Zanconato et al., 2015).

### Mechanisms controlling YAP cytoplasmic localisation in differentiating daughter cells of squamous versus columnar epithelia

The above analysis suggests that daughter cells differentiate in the skin simply by loss of contact with the basement membrane extracellular matrix and consequent loss of integrin-Src signalling, EGFR-PI3K signalling and YAP nuclear localisation. This model is plausible in all stratified squamous epithelia, but cannot explain the self-renewal versus differentiation decision in columnar epithelia because differentiated columnar epithelial cells retain contact with the basement membrane. Thus, columnar cells must employ an additional mechanism to promote YAP localisation to the cytoplasm. An obvious candidate is the expression of a differentiated apical plasma membrane domain in columnar epithelial cells, because apical proteins associated with Crumbs (CRB3) are well known to induce Hippo signalling (MST-LATS signalling) to drive YAP to the cytoplasm (Chen et al., 2010; Fletcher et al., 2015; Ling et al., 2010; Szymaniak et al., 2015; Varelas et al., 2010).

**Fig. 6.**
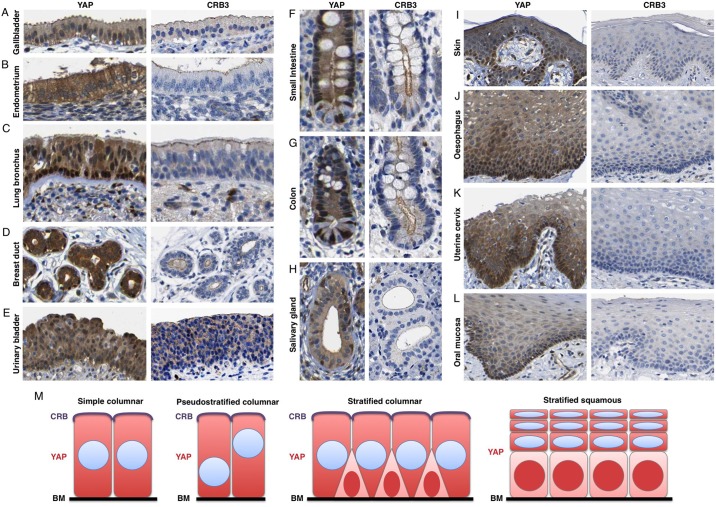
**Apical-domain formation inhibits YAP nuclear localisation in human columnar epithelia.** The Human Protein Atlas dataset was mined to compare the localisation of YAP with the presence of absence of the apical domain in different epithelia. YAP localises to the cytoplasm in columnar gallbladder epithelium (A) and columnar endometrial epithelium (B) which have a CRB3^+^ apical domain. (C) YAP localises to the nucleus of basal layer stem/progenitors, which lack CRB3 expression, and cytoplasm in columnar epithelial cells, which have a CRB3^+^ apical domain, in the bronchus. (D) YAP localises to the nucleus of basal layer stem/progenitors, which lack CRB3 expression, and cytoplasm in columnar epithelial cells, with a CRB3^+^ apical domain, in the breast. (E) YAP localises to the cytoplasm in pseudostratified columnar bladder epithelium, with a CRB3^+^ apical domain. (F) YAP localises to the nucleus of crypt base stem/progenitors, which lack a large CRB3^+^ apical domain, and cytoplasm in columnar epithelial cells, which feature a large CRB3^+^ apical domain, in the small intestine. (G) YAP localises to the nucleus of crypt base stem/progenitors, which have a small CRB3^+^ apical domain, and cytoplasm in columnar epithelial cells, which feature a large CRB3^+^ apical domain, in the colon. (H) YAP localises to the nucleus of basal layer stem/progenitors, which lack CRB3 expression, and cytoplasm in columnar epithelial cells, which have a CRB3^+^ apical domain, in the salivary gland. (I-L) YAP localises to the nucleus of basal layer stem/progenitors, and cytoplasm of differentiating squamous epithelial cells, even though the entire tissue lacks CRB3 expression. (M) Schematic diagram of YAP localisation in different epithelial tissue types.

To test this notion *in vivo*, we compared the subcellular localisation of YAP with that of CRB3 in columnar epithelia in the gallbladder, endometrium, lung bronchus, breast duct, urinary bladder, small intestine, colon and salivary gland. In all cases, apical localisation of CRB3 in differentiated daughter cells correlated with cytoplasmic localisation of YAP (Fig. 6A-H). Accordingly, basal layer stem/progenitor cells of the lung, breast or intestine retained nuclear YAP but lack apical CRB3. Notably, CRB3 is not expressed in squamous epithelia so cannot mediate the regulation of YAP in these tissues (Fig. 6I-L). The key Hippo pathway components Merlin, SAV1 and KIBRA colocalised with CRB3 (Figs S4 and S5) (Chen et al., 2010; Genevet et al., 2010; Hamaratoglu et al., 2006; Ling et al., 2010; Yin et al., 2013; Yu et al., 2010; Zhang et al., 2010). These results indicate that a universal regulatory logic exists in which YAP nuclear localisation requires contact with the basement membrane but is inhibited by expression of an apical domain (Fig. 6M).

**Fig. 7.**
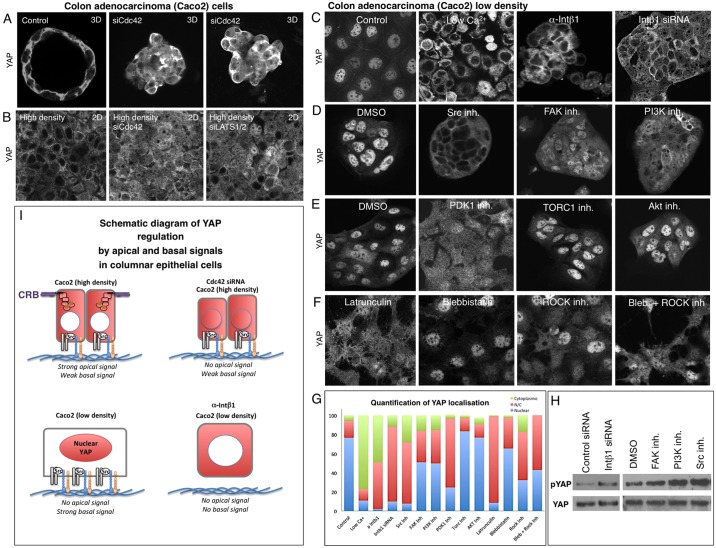
**Basal integrin-Src signalling promotes YAP nuclear localisation in human Caco2 epithelial cells when apical domain formation is blocked.** (A) Caco2 colon adenocarcinoma cells form 3D cysts in cell culture that feature cytoplasmic YAP localisation. Silencing of *CDC42* by siRNA knockdown disrupts apical-basal polarity and induces more nuclear YAP localisation. (B) Caco2 colon adenocarcinoma cells form 2D epithelial monolayers at high density. Silencing of *CDC42* by siRNA knockdown disrupts apical-basal polarity and induces more nuclear YAP localisation, similar to silencing of *LATS1/2*. (C) YAP nuclear localisation is very strong when Caco2 cells are plated at low density to prevent apical domain formation. Nuclear localisation is prevented by treatment of Caco2 cells with low-calcium medium, anti-ITGB1 antibodies (PD52) or by *ITGB1* siRNA treatment, but not in controls. (D) YAP nuclear localisation is prevented by treatment of Caco2 cells with the Src inhibitor Dasatinib, by the FAK inhibitor PF573228 or by the PI3K inhibitor GDC0941, but not by treatment with DMSO solvent. (E) YAP nuclear localisation is reduced by treatment of Caco2 cells with the PDK1 inhibitor BX795, but not by the AKT inhibitor MK2206, TORC1 inhibitor Everolimus or DMSO solvent. (F) YAP nuclear localisation is reduced by treatment of Caco2 cells with the F-actin destabilising drug Latrunculin, the myosin II inhibitor Blebbistatin or the Rho kinase inhibitor Y27632, or a combination of Blebbistatin and Y27532. (G) Quantification of C-F. (H) Western blotting analysis of p-YAP levels in Caco2 cells treated with control siRNAs or *ITGB1* siRNAs, as well as DMSO control, FAK inhibitor, PI3K inhibitor or Src inhibitor. Total YAP levels are shown as a control. (I) Schematic diagram of YAP regulation in Caco2 cells.

We next sought to confirm that apical and basal signals act antagonistically in columnar epithelial cells. We examined human intestinal epithelial cells in culture that are capable of forming 3D cysts or 2D monolayers in which YAP becomes cytoplasmic. We found that siRNA knockdown of the apical determinant CDC42 or LATS1/2 has similar effects, driving YAP to the nucleus (Fig. 7A,B). Strong YAP nuclear localisation can also be achieved simply by plating these cells at low density, so that they are unable to differentiate an apical domain and also retain a flat morphology with an extensive basal surface area (Fig. 7C). This basal contact appears to invoke the same integrin-Src signals identified in keratinocytes, because blocking of integrins with low Ca^2+^, anti-ITGB1 antibodies or *ITGB1* siRNAs relocalised YAP to the cytoplasm (Fig. 7C). Inhibition of Src, FAK, PI3K or PDK1 also impaired YAP nuclear localisation (Fig. 7D,E). These effects are once again as strong as inhibition of F-actin, myosin II or Rho kinase (Fig. 7F,G). Examination of p-YAP levels indicated that integrin-Src signalling acts via regulation of MST-LATS phosphorylation of YAP (Fig. 7H). These results suggest that apical domain formation activates LATS kinases to retain YAP in the cytoplasm, whereas basal integrin-Src and PI3K signalling inhibits LATS kinases to promote nuclear YAP localisation (Fig. 7I).

**Fig. 8.**
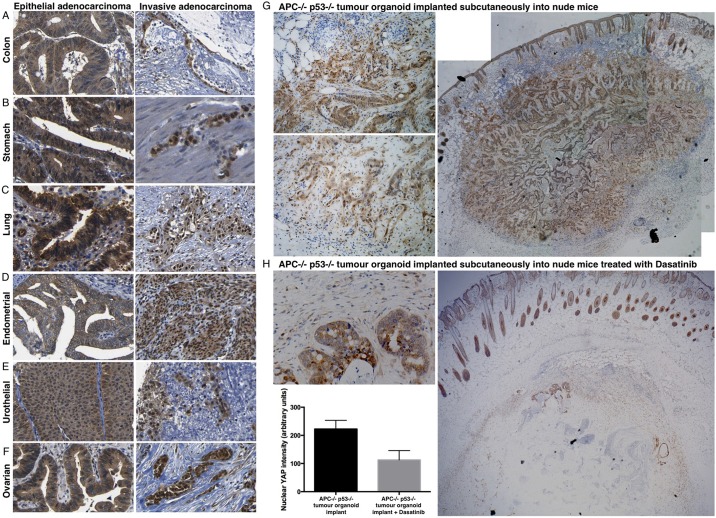
**YAP becomes nuclear in invasive adenocarcinomas, which become sensitive to Dasatinib.** In the colon (A) and stomach (B) YAP localises to the cytoplasm of columnar epithelial cells in epithelial adenocarcinoma, and the nucleus of invasive adenocarcinoma cells, which have lost their columnar shape and lack a lumen. (C) In the bronchus, YAP localises to the nucleus of basal layer stem/progenitors and cytoplasm in columnar epithelial cells in epithelial adenocarcinoma, and the nucleus of invasive adenocarcinoma. (D) In the endometrial epithelium, YAP localises to the cytoplasm of columnar epithelial cells in epithelial adenocarcinoma, and the nucleus of invasive adenocarcinoma cells. (E) In urothelial epithelium, YAP localises to the cytoplasm in pseudostratified columnar cells in epithelial adenocarcinoma and to the nucleus of invasive adenocarcinoma. (F) YAP localises to the cytoplasm in ovarian adenocarcinoma, and to the nucleus of invasive ovarian adenocarcinoma. (G) YAP staining in *Apc^−/−^ p53^−/−^* tumour organoids implanted subcutaneously into nude mice, which invade dramatically into the surrounding tissue. Note that cells at the invasive front feature nuclear YAP localisation, whereas columnar epithelial cells in the central regions of the tumour feature cytoplasmic YAP localisation. (H) YAP staining is strongly reduced by Dasatinib treatment of *Apc^−/−^ p53^−/−^* tumour organoids implanted subcutaneously into nude mice. Invasive tumour cells are not visible. Quantification of YAP nuclear localisation was performed on *n*=200 tumour cells from G and H. Values are means±s.e.m.

To further explore these findings *in vivo*, we examined how YAP behaves in columnar epithelial tumours that progress to invasive adenocarcinomas. We found that YAP remains cytoplasmic whereas tumours of the colon, stomach, lung, endometrium, urothelium or ovary retained their columnar epithelial form (Fig. 8A-F). By contrast, invasive adenocarcinomas of the same tissue origin all featured a loss of columnar form and a dramatic localisation of YAP to the nucleus (Fig. 8A-F). These results suggest that loss of the apical domain during tumour progression allows YAP to become nuclear. We therefore tested whether nuclear YAP in invasive adenocarcinomas would be sensitive to inhibition of integrin-Src signalling with Dasatinib. We examined *Apc*^−/−^ *p53* (*Trp53*)^−/−^ mutant intestinal organoids that had been implanted subcutaneously into nude mice. On transplantation, these organoids rapidly produce highly invasive adenocarcinomas, entering the surrounding stromal tissue. We found that YAP localisation became strongly nuclear, specifically in the invasive tumour cells (Fig. 8G). We next treated mice carrying such invasive tumours with the Src inhibitor Dasatinib, which strongly suppressed nuclear YAP localisation and reduced tumour growth and invasion (Fig. 8H). These findings indicate that Src activity promotes YAP nuclear localisation *in vivo* and suggest a potential therapy for invasive adenocarcinomas and carcinomas.

## DISCUSSION

Our results identify a physiological role for YAP and TAZ in skin homeostasis, promoting cell proliferation in basal layer stem/progenitor cells (Figs 1, 2). YAP and TAZ localise to the nucleus of basal layer cells to drive transcription of a set of genes associated with cell cycle progression, cell growth, EGFR signalling and cell-matrix adhesion via integrins. In the absence of YAP and TAZ, proliferation is reduced and dramatic hair loss occurs, indicating that YAP and TAZ are crucial players in the stem/progenitor cell biology of the skin. Importantly, loss of either YAP or TAZ individually had no visible phenotype, confirming that the two proteins act in a redundant fashion in this tissue.

Both YAP and TAZ localise to the nucleus in the basal layer cells of the skin and we focused on YAP to characterize the molecular mechanisms responsible for this nuclear localisation (Figs 3–5). We examined the model that integrin-Src and EGFR-PI3K signalling promotes YAP nuclear localisation, which was first proposed based on experiments in MCF10A breast cells in culture (Fan et al., 2013; Kim and Gumbiner, 2015), and found that these signalling molecules are indeed strongly expressed in basal layer skin cells and are essential to promote YAP nuclear localisation in keratinocytes in culture and in mouse basal layer skin cells *in vivo*. Since YAP appears to induce expression of integrins, integrin ligands (CYR61, CTGF) and EGFR ligands (AREG), we propose that a positive feedback loop drives basal layer stem/progenitor cell identity and that this loop is broken when daughter cells lose contact with the basement membrane and differentiate – forming a bistable system of cell fate determination. Our findings provide an explanation for how these signalling pathways integrate to control skin stem/progenitor cell biology.

The notion that nuclear localisation of YAP occurs upon contact with basement membrane extracellular matrix applies equally to other squamous epithelia. By contrast, columnar epithelia differentiate an apical domain that induces YAP relocalisation to the cytoplasm via apical CRB3-MER-KIBRA-SAV signals, which are known to activate the MST and LATS kinases to promote YAP phosphorylation and cytoplasmic retention despite contact with the basement membrane (Figs 6, 7, Figs S4-S6) (Baumgartner et al., 2010; Chen et al., 2010; Fletcher et al., 2015; Genevet et al., 2010; Hamaratoglu et al., 2006; Ling et al., 2010; Sun et al., 2015; Szymaniak et al., 2015; Varelas et al., 2010; Yin et al., 2013; Yu et al., 2010). The CRB3-MER-KIBRA-SAV complex appears to be absent in squamous epithelia, which never differentiate a true apical domain, leaving basement membrane contact as the sole regulatory mechanism (Fig. 6). Thus, our results show that antagonistic apical and basal polarity signals serve as the primary control mechanism that determines YAP subcellular localisation *in vivo*. In a striking parallel, the same apical and basal polarity determinants act antagonistically in epithelial membrane polarisation, with integrin and PI3K signalling localising PtdIns(3,4,5)P3 basally, and helping to restrict and/or orient PtdIns(4,5)P2, CDC42 and the CRB3 complex apically (Akhtar and Streuli, 2013; Bryant et al., 2014; Fletcher et al., 2012; Martin-Belmonte et al., 2007; Thompson, 2013). Further work will be necessary to fully elaborate the molecular interactions that mediate the antagonistic relationship between apical and basal signals in cell polarisation and nuclear signalling via YAP.

This fundamental control mechanism appears to be conserved across the animal kingdom. For example, *Drosophila* simple columnar epithelia such as imaginal discs rely primarily on apical Crb-Mer/Ex-Kibra-Sav signalling to retain Yki in the cytoplasm and restrict tissue growth (Baumgartner et al., 2010; Chen et al., 2010; Genevet et al., 2010; Hamaratoglu et al., 2006; Ling et al., 2010; Yu et al., 2010). By contrast, *Drosophila* stratified columnar epithelia such as the intestine require integrins, Src, EGFR and Yki to promote proliferation of basal layer stem/progenitor cells, suggesting that the regulatory connection between them described here is also conserved (Cordero et al., 2014; Jiang et al., 2011; Kohlmaier et al., 2015; Lin et al., 2013; Shaw et al., 2010; Staley and Irvine, 2010; Xu et al., 2011). Furthermore, ectopic activation of Src, EGFR, PI3K or Yki in simple columnar imaginal discs is sufficient to induce overproliferation of cells, whereas loss of PI3K or Yki strongly impairs imaginal disc tumour formation (Doggett et al., 2011; Enomoto and Igaki, 2013; Fernandez et al., 2014; Herranz et al., 2012, 2014; Strassburger et al., 2012; Willecke et al., 2011).

Our model raises interesting questions about the possible physiological roles of other YAP regulators identified in cell culture, namely that YAP is controlled by Wnt signalling (Azzolin et al., 2014; Cai et al., 2015; Park et al., 2015), GPCR signalling (Yu et al., 2012), PKA signalling (Yu et al., 2013), LKB1-MARK signalling (Mohseni et al., 2014), protease-activated receptors (Mo et al., 2012) or the Mevalonate pathway (Sorrentino et al., 2014). Further work is necessary to understand in which tissues and under what conditions these diverse signals are utilised and integrated *in vivo*.

Importantly, our model is easily reconciled with a possible role of YAP as a mechanosensor in epithelial tissues *in vivo* (Dupont et al., 2011). Mechanical force has been proposed to modulate signalling by both the apical Crb-Mer/Ex-Kibra-Sav system in *Drosophila* (Fletcher et al., 2015; Rauskolb et al., 2014) as well as the basal integrin-Src system in mammalian cell culture (reviewed in Humphrey et al., 2014; Lawson and Burridge, 2014). In the early mouse pre-implantation embryo, cortical tension is higher in outer cells than inner cells, leading to nuclear YAP in outer cells despite the presence of an apical domain (Anani et al., 2014; Kono et al., 2014; Nishioka et al., 2009). Consistently, reducing cortical tension with a ROCK inhibitor abolishes nuclear localisation of YAP in early mouse embryos (Anani et al., 2014; Kono et al., 2014; Nishioka et al., 2009). Mechanical forces might also explain why YAP becomes nuclear in some terminally differentiating and extremely flattened keratinocytes (Fig. 5, cells marked by asterisk).

Finally, our model is also easily reconciled with the ability of YAP to respond to inflammatory cues in epithelia, such as interleukin-6 (IL-6). Recent work revealed that the IL-6 co-receptor gp130 triggers stabilisation and nuclear translocation of YAP via Src kinases (Taniguchi et al., 2015). This signalling module was shown to be activated by mucosal injury to intestinal epithelia to promote intestinal regeneration, a known Src and YAP function (Cai et al., 2010; Cordero et al., 2014; Taniguchi et al., 2015). We confirm that tissue damage (with 14Gy of radiation) elevates YAP levels in a Src-dependent manner in the intestine (Fig. S7). However, we note that upon damage, localisation of YAP is still mostly nuclear in basal crypt stem cells, which normally lack an apical domain, and mostly cytoplasmic in differentiated columnar epithelial cells, which have an apical domain (Fig. S7). Interestingly, hyperproliferation per se driven by conditional deletion of APC and oncogenic mutation of KRAS (*VillinCreER Apc^fl/fl^ K-ras^LSL-G12D^*) does not change the fundamental pattern of YAP localisation, with localisation of YAP remaining most strongly nuclear in the basal stem cells but not the columnar epithelial cells (Fig. S6). In the skin, YAP is also elevated upon wounding (Fig. 2C,D) or inflammation (upon TPA treatment) in a Src-dependent manner (Fig. 5), but remains most strongly nuclear in the basal layer stem/progenitor cell population (Fig. 5). Thus, Src acts as a point of convergence between inflammatory cues and apical-basal polarity cues, with polarity cues being the dominant input. Further work is necessary to understand whether Src acts primarily by directly phosphorylating YAP or indirectly via enhancing PI3K signalling to inhibit MST-LATS activity. Nevertheless, our findings add weight to the notion that chemical inhibitors of Src kinases such as Dasatinib are promising cancer therapeutics (Creedon and Brunton, 2012; Karim et al., 2013) (Fig. 8).

In conclusion, epithelial stem/progenitor cell proliferation and differentiation might be regulated primarily by apical-basal polarity signals. In particular, YAP, a key driver of cell proliferation in stem/progenitor cells and cancer, appears to act primarily as a sensor of epithelial cell polarity and only secondarily as a sensor of other stimuli. Stem/progenitor cells thus use information about their polarity status to inform their decisions to either proliferate or arrest/differentiate via control of YAP. In the skin epithelium, nuclear YAP acts redundantly with TAZ to drive gene expression in the basal stem/progenitor cell layer to maintain cell proliferation and normal tissue homeostasis.

## MATERIALS AND METHODS

### Mouse strains

All experiments were carried out in accordance with the United Kingdom Animal Scientific Procedures Act (1986) and UK Home Office regulations under project licence number 70/7926. The *Yap^fl/fl^ Taz^fl/fl^* mice were a gift from Axel Behrens (Francis Crick Institute). *K5-CreERt* mice were obtained from Ian Rosewell (Francis Crick Institute). *v-HA-Ras* transgene (TG.AC) mice were a gift from Ilaria Malanchi (Francis Crick Institute) and have been previously described (Leder et al., 1990). Wild-type mice were used in mixed background. All transgenic mice were in mixed background and used with littermate controls. APC p53 tumour sections from implanted nude mice were obtained from Owen Sansom (The Beatson Institute). *K14-Cre Fak^fl/fl^* mice and *Src^fl/fl^, Fyn^−/−^, Yes^−/−^* mice were obtained from Val Brunton (University of Edinburgh) and were described previously (Marcotte et al., 2012; Ridgway et al., 2012). *Apc^−/−^ p53^−/−^* (*Apc^580D/580D^ P53 Trp53Δ^2−10^* allele) mice were obtained from Owen Sansom and were previously described (Jonkers et al., 2001; Shibata et al., 1997). *AhCre* is previously described (Ireland et al., 2004). *K-ras^G12D^* allele is from Tyler Jacks (Jackson et al., 2001).

### siRNA treatment

Human Caco-2, A431 or HaCAT cells were cultured as previously stated (Fletcher et al., 2015; Elbediwy et al., 2012). All siRNA transfections were performed using Lipofectamine RNAiMax transfection reagent (Invitrogen). Briefly, cells were seeded in 6-well plates and treated with the siRNA/transfection mix 2 h post seeding. A final concentration of 50-100 nM siRNA was used for transfections. The following day, another transfection was performed before the cells were trypsinised 4 h later and reseeded either for 2D or 3D culture. 2D siRNA treatments were left for a total of 72 h and 3D treatments were left for a total of 120 h. 3D cultures were prepared as previously stated (Elbediwy et al., 2012). siRNAs were used as siGenome pools (Dharmacon).

### Treatment with inhibitor, blocking antibody and low-calcium medium

2D mammalian inhibitor treatments were for 4 h. They were as follows: 5 µM PF573228 (FAK); 5 µM Saracatinib (Src); 5 µM Dasatinib (Src/Abl); 5 µM BX795 (PDK1); 5 µM MK2206 (AKT); 2 µM GDC0941 (PI3K); 100 µM Blebbistatin (myosin); 100 µM Y27632 (Rock); 2 µM Latrunculin A (actin) and 3 µM Everolimus (mTOR); reagents were supplied by Sigma-Aldrich and Stratech Scientific Ltd. Integrin β1 blocking antibody or control IgG antibody (gifts from Nancy Hogg, Francis Crick Institute) was incubated with the cells for 1 h at a concentration of 10 µg/ml before the cells were replated. Low-calcium conditions were as previously reported (Elbediwy et al., 2012). 2D wound healing involved plating the cells at high density, causing a scratch and subsequent addition of Dasatinib for 4 h.

### Antibodies, image acquisition and quantification

Primary antibodies used were: rabbit YAP (H-125, Santa Cruz, sc-15407; 1:200IF, 1:1000 IB), mouse YAP (63.7; Santa Cruz, sc-101199; 1:200 IF, 1:1000 IB), rabbit p-YAP (Cell Signaling Technology, 4911; 1:1000 IB). Samples were imaged with a Leica SP5 confocal microscope using a 63× oil immersion objective and processed using Adobe Photoshop. Fixation and cell culture quantification was carried out as previously described (Fletcher et al., 2015).

### YAP/TAZ conditional deletion

Tamoxifen (Sigma, 20 mg/ml in peanut oil) was injected intraperitoneally (IP) (5 µl/g body weight) for 5 consecutive days into 8- to 16-week-old controls or transgenic animals carrying *K5-CreERt Yap^fl/fl^ Taz^fl/fl^* to induce Yap/Taz knockdown and analysed for Yap/Taz deficiency by immunohistochemistry 7 days thereafter. *K5-CreERt Yap^fl/fl^ Taz^fl/fl^* mice used for long-term analysis were subsequently IP injected with tamoxifen every month for 3 consecutive days and analysed 8 weeks after the start of tamoxifen treatment.

### Wound healing

Following the 5-day tamoxifen treatment, 4 hydroxy-tamoxifen (4OHT, Sigma) was topically applied to shaved back skin for 5 consecutive days at a dosage of 10 mg/ml in ethanol and 100 µl was applied per mouse. Mice were anaesthetised with IsoFlo (Isoflurane, Abbott Animal Health) and treated with the analgesics Vetergesic (Alstoe Animal Health) and Rimadyl (Pfizer Animal Health) for 2 days after wounding. A 4 mm punch wound was made in the back skin using a biopsy punch (Miltex) 10 days after tamoxifen/4OHT treatment start and wound closure monitored over time.

### Dasatinib treatment of skin

Wild-type mice between 8 and 12 weeks of age were topically treated with 150 µl Dasatinib (10 µM in DMSO, Selleck) onto the shaved back skin directly followed by 200 µl TPA (12-O-tetradecanoylphorbol-13-acetate; stock dissolved in DMSO and diluted in acetone, 12.5 µg/mouse) treatment for 2 consecutive days. Mice were analysed and the back skin harvested on the third day. Control mice were treated with DMSO and acetone.

### Chemical carcinogenesis

Chemical skin carcinogenesis was induced on 12-week-old *v-Ha-Ras* transgene (TG.AC)-expressing mice in mixed background by a single application of 100 µg/mouse DMBA [7,12-dimethylbenz(a)anthracene, Sigma] onto the shaved backskin followed by biweekly topical treatments with TPA (4 µg/mouse) starting 1 week after DMBA application. Skin papillomas were detectable 8 weeks after start of DMBA-TPA treatment and harvested at 13 weeks. For Dasatinib treatment, papillomas allowed to reach ∼1 cm^3^. These established papillomas were treated topically with Dasatinib (10 µM in DMSO/acetone; 100 µl/papilloma) once and analysed 3 or 7 days thereafter. For generation of skin carcinomas, ∼12-week-old DMBA-treated FVB/N wild-type mice were treated biweekly with 4 µg/mouse TPA onto shaved back skin for 10 weeks then weekly for a further 4 weeks before the carcinomas were harvested.

### Intestinal experiments

Mice carrying the *AhCre* recombinase were induced by three intraperitoneal (i.p.) injections of 80 mg/kg β-Napthoflavone for 1 day. Intestinal phenotypes were analysed 4 or 7 days after transgene induction to assess homeostasis or regeneration, respectively. Intestinal regeneration was induced by irradiating mice with 14Gy gamma-irradiation 4 days after recombinase induction. Mice were sacrificed 72 h post irradiation and the small intestine isolated and flushed with tap water. Ten 1 cm portions of small intestine were bound together with surgical tape and fixed in 4% neutral buffered formalin.

### Organoid transplantation experiments

Intestinal crypts from *VillinCreER Apc^fl/fl^ p53^fl/fl^* mice were removed 4 days following Cre induction with Tamoxifen (2 mg). This causes full recombination at both the *Apc* and *p53* loci and organoids now grow as spheres in an R-Spondin-independent manner (Sato et al., 2009). For transplantation of organoids, 50 organoids were transplanted subcutaneously into nude mice (see Valeri et al., 2014). A dose of 10 mg/kg Dasatinib daily gavage was chosen as we have previously shown to cause a reduction in p-SRC *in vivo* without toxicity (Morton et al., 2010). Mice were treated continuously from 10 days post injection of spheres.

### Immunohistochemistry

Mouse back skin samples were harvested and fixed in neutral-buffered formaldehyde 10% v/v and then embedded in paraffin blocks. 4-µm-thick sections were cut, deparaffinised and rehydrated using standard methods. After an antigen retrieval step, sections were stained with Hematoxylin and Eosin (H&E) solution or with primary antibody. Additional images of human samples were obtained by datamining the www.proteinatlas.org database (Berglund et al., 2008; Lundberg and Uhlén, 2010; Pontén et al., 2008; Uhlen et al., 2005, 2015, 2010).

### RNA-seq analysis

A431 or HaCAT cell lysates transfected with empty vector, YAP1 S5A, control siRNA or YAP1 siRNAs were used. Sequencing was performed on biological triplicates on the Illumina HiSeq 2500 platform and generated ∼69 million 100 bp paired end reads per sample (data deposited in GEO under accession number GSE80082). Sequenced reads were trimmed to 75 base pairs and mapped to the Refseq genome model, using RSEM (v.1.2.21). RSEM uses the bowtie2 alignment tool. Gene counts were filtered to remove genes with 10 or fewer mapped reads per sample. TMM (treated mean of M-values) normalisation and differential expression analysis using the negative binomial model was carried out with the R-Bioconductor package ‘EdgeR’. Genes with logCPM>1 and FDR<0.05 were judged to be differentially expressed. Enrichments of pathway, category and motif gene sets were assessed using GSEA with logFC pre-ranked gene lists. Gene sets with an enrichment false discovery rate (FDR) value of less than 0.05 were judged to be strongly statistically significant and values of less than 0.25 significant.

### qPCR

Total RNA was extracted from homogenised mouse skin using an RNeasy Mini Kit (Qiagen). cDNA synthesis for WT or dKO mice was performed using Superscript II (Invitrogen). Primers were purchased as Quantitect Primers (Qiagen). Gene samples were run in triplicate on a Quantstudio 12 Flex Thermocycler. Expression values were calculated using the ΔΔCT method relative to the housekeeping gene β-2-microglobulin (B2M). All error bars indicate s.e.m.
